# Impact of Synchronous Versus Metachronous Onset of Colorectal Peritoneal Metastases on Survival Outcomes After Cytoreductive Surgery (CRS) with Hyperthermic Intraperitoneal Chemotherapy (HIPEC): A Multicenter, Retrospective, Observational Study

**DOI:** 10.1245/s10434-019-07294-y

**Published:** 2019-03-15

**Authors:** Judith E. K. R. Hentzen, Koen P. Rovers, Hendrien Kuipers, Willemijn Y. van der Plas, Lukas B. Been, Frederik J. H. Hoogwater, Robert J. van Ginkel, Patrick H. J. Hemmer, Gooitzen M. van Dam, Ignace H. J. T. de Hingh, Schelto Kruijff

**Affiliations:** 1 Department of Surgery, Division of Surgical Oncology, University Medical Center Groningen, University of Groningen, Groningen, The Netherlands; 2 Department of Surgery, Division of Surgical Oncology, Catharina Hospital Eindhoven, Eindhoven, The Netherlands; 3 Department of Surgery, Division of Hepatopancreatobiliary Surgery and Liver Transplantation, University Medical Center Groningen, University of Groningen, Groningen, The Netherlands; 4 Department of Nuclear Medicine and Molecular Imaging and Intensive Care, University Medical Center Groningen, University of Groningen, Groningen, The Netherlands; 5 GROW, School for Oncology and Developmental Biology, Maastricht University, Maastricht, The Netherlands

## Abstract

**Background:**

Careful selection of patients with colorectal peritoneal metastases (PM) for cytoreductive surgery (CRS) with hyperthermic intraperitoneal chemotherapy (HIPEC) is crucial. It remains unknown whether the time of onset of colorectal PM (synchronous vs metachronous) influences surgical morbidity and survival outcomes after CRS with HIPEC.

**Methods:**

Patients with histologically proven colorectal PM who underwent CRS with HIPEC between February 2006 and December 2017 in two Dutch tertiary referral hospitals were retrospectively included from a prospectively maintained database. The onset of colorectal PM was classified as synchronous (PM diagnosed at the initiational presentation with colorectal cancer) or metachronous (PM diagnosed after initial curative colorectal resection). Major postoperative complications (Clavien–Dindo grade ≥ 3), overall survival (OS), and disease-free survival (DFS) were compared between patients with synchronous colorectal PM and those with metachronous colorectal PM using Kaplan–Meier analyses, proportional hazard analyses, and a multivariate Cox regression analysis.

**Results:**

The study enrolled 433 patients, of whom 231 (53%) had synchronous colorectal PM and 202 (47%) had metachronous colorectal PM. The major postoperative complication rate and median OS were similar between the patients with synchronous colorectal PM and those with metachronous colorectal PM (26.8% vs 29.7%; *p* = 0.693 and 34 vs 33 months, respectively; *p* = 0.819). The median DFS was significantly decreased for the patients with metachronous colorectal PM and those with synchronous colorectal PM (11 vs 15 months; adjusted hazard ratio, 1.63; 95% confidence interval, 1.18–2.26).

**Conclusions:**

Metachronous onset of colorectal PM is associated with early recurrence after CRS with HIPEC compared with synchronous colorectal PM, without a difference in OS or major postoperative complications. Time to onset of colorectal PM should be taken into consideration to optimize patient selection for this major procedure.

**Electronic supplementary material:**

The online version of this article (10.1245/s10434-019-07294-y) contains supplementary material, which is available to authorized users.

Colorectal cancer (CRC) is one of the most common cancers worldwide, with 1.4 million new cases and more than 700,000 deaths per year.^1^ Approximately 30– 40% of CRC patients experience peritoneal metastases (PM) at some point in time after the initial diagnosis.^2^–^7^ With the systemic therapy regimens, the median overall survival (OS) for patients with colorectal PM traditionally ranges from 12 to 24 months.^8^–^10^

Almost three decades ago, a curative-intent treatment option arose: cytoreductive surgery (CRS) combined with hyperthermic intraperitoneal chemotherapy (HIPEC).^11^^,^^12^ The main principle of this extensive procedure is removal macroscopic disease during CRS, followed by HIPEC for microscopic malignant tissue, resulting in an OS of up to 5 years for highly selected patients with colorectal PM.^11^–^13^ However, CRS with HIPEC is accompanied by substantial early recurrence rates (up to 50% during the first year after treatment), morbidity (16–64%), and mortality (0–8%).^14^–^20^ Therefore, careful patient selection is pivotal to prevention of early recurrence and therefore overtreatment, with the aim to increase survival and reduce morbidity and mortality.

At this writing, the most powerful prognostic factors for survival after CRS with HIPEC are extent of disease measured by the Peritoneal Cancer Index (PCI), completeness of the performed cytoreduction, and signet ring cell histology.^21^–^27^ These prognostic factors, on which surgeons rely heavily, are determined during or after the surgical procedure rather than in a preoperative setting. Therefore, more research on preoperative prognostic factors is of utmost importance to improvement of the decision-making process.

The development of PM metachronously or synchronously with the primary CRC diagnosis might be of relevance. The difference in either tumor biology and behavior or adequate initial treatment might influence OS and DFS. In an attempt to discover novel preoperative risk factors for worse outcomes, this study aimed to investigate the impact of the synchronous versus the metachronous onset of colorectal PM on surgical morbidity and survival outcomes after CRS with HIPEC.

## Methods

### Design, Setting, and Participants

In this multicenter observational study, data for all consecutive patients with histologically proven colorectal PM who underwent CRS with HIPEC between February 2006 and December 2017 were retrospectively extracted from a merged prospectively maintained institutional database of two Dutch tertiary referral hospitals.

**Fig. 1:**
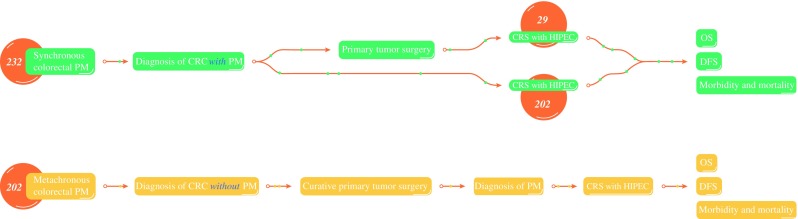
Definitions of synchronous and metachronous colorectal peritoneal metastases. Synchronous colorectal peritoneal metastases are peritoneal metastases diagnosed at the patient’s initial presentation with colorectal cancer. Metachronous colorectal peritoneal metastases are peritoneal metastases diagnosed after initial curative colorectal resection

No worldwide consensus exists concerning the definitions of the synchronous and metachronous formations of peritoneal metastases. The most common definitions used in scientific literature were selected. Patients with synchronous colorectal PM had colorectal cancer diagnosed at the time of presentation, either on routine staging, on computed tomography (CT), or at laparotomy. Patients with metachronous colorectal PM were deemed to be clear of peritoneal disease at the initial “curative” colorectal resection, but subsequently became symptomatic during the follow-up period and had PM diagnosed on computed tomography (CT) (Fig. 1). The study was approved by the Institutional Ethics Committee of the University Medical Center Groningen (METc 201800395).

### Preoperative Evaluation and Management

All the patients underwent a standardized preoperative workup to evaluate eligibility for CRS with HIPEC, with the aim of achieving complete cytoreduction with acceptable risk of treatment-related morbidity and mortality. This preoperative workup consisted of a clinical examination, preoperative laboratory testing, and thoracic, abdominal, and pelvic CT with oral and intravenous contrast agents to quantify the peritoneal disease burden and rule out extra-abdominal metastases. If deemed necessary, a diagnostic laparoscopy (DLS) was performed to assess the location and extent of peritoneal disease using the PCI scoring system, as described by Sugarbaker et al.^28^ Clinically suspect lesions during DLS were biopsied for pathologic confirmation of colorectal PM.

Next, the eligibility for CRS with HIPEC according to the preoperative workup was determined for each patient at a multidisciplinary oncology team meeting. In the Netherlands, candidates for CRS with HIPEC are generally those with colorectal PM amenable to complete cytoreduction, a PCI below 20, no extra-abdominal metastases, and a performance status that allows for major surgery. The presence of up to three resectable liver metastases is not an absolute contraindication for CRS with HIPEC.^17^

### Cytoreductive Surgery with HIPEC

For the patients in this study, CRS was performed only if the colorectal PM was deemed to be completely resectable after exploratory laparotomy, whereas HIPEC was performed only in case of a (near) complete cytoreduction. The two institutions performed CRS with HIPEC under the same standardized Dutch HIPEC protocol, as previously described.^17^ Restrictions were imposed on the extent of surgery as far as it was compatible with sufficient postoperative function. At the end of surgery, the Completeness of Cytoreduction (CC) score was determined,^28^ with CC-0 indicating that no residual tumor was visible or palpable in the peritoneal cavity, CC-1 indicating residual tumor deposits smaller than 2.5 mm, CC-2 indicating residual tumor deposits between 2.5 mm and 2.5 cm, and CC-3 indicating residual tumor deposits above 2.5 cm or a confluence of nodules.

The HIPEC procedure was then performed by circulating a heated solvent infused with chemotherapeutic medication throughout the abdomen using the open-colosseum technique.^29^ In most cases, mitomycin (35 mg/m^2^) was administered in the open abdominal cavity with a temperature of 41–42 °C for 90 min. After this, the fluid was evacuated from the abdomen, and the continuity of the gastrointestinal tract was restored. After surgery, patients were admitted to the intensive care unit for at least one postoperative day until both cardiac and pulmonary functions were stable.

### Follow-Up Evaluation

All the patients were followed by a standardized follow-up protocol. Physical examination and carcinoembryonic antigen (CEA) measurements were performed on a 3- to 6-month basis for a minimum of 4 years. If recurrence of the disease (e.g., clinical symptoms or increase in CEA levels) was suspected, a CT of the thorax and abdomen was performed, with tissue biopsies in selected cases.

### Data Collection

Data on patient characteristics, tumor characteristics, operative characteristics, postoperative morbidity and mortality, recurrence, and overall survival were collected prospectively. Data on postoperative complications were collected up to 60 days after CRS with HIPEC and registered according to the Clavien–Dindo classification system.^30^

Data regarding the use of perioperative chemotherapy were divided into three categories. Chemotherapy before CRS with HIPEC was recorded as “neoadjuvant chemotherapy.” Chemotherapy after CRS with HIPEC was recorded as “adjuvant chemotherapy,” and when chemotherapy was used in the past (e.g., before or after a primary colorectal tumor resection), it was recorded as “prior chemotherapy.” Data were collected and stored in compliance with the Declaration of Helsinki.

### Primary and Secondary Outcomes

The primary outcome was overall survival (OS), defined as the time between CRS with HIPEC and death or the date of the last follow-up visit in censored cases. The secondary outcomes were disease-free survival (DFS) and major postoperative complications. In this study, DFS was defined as the time between CRS with HIPEC and the date of the first recurrence or the last follow-up visit in censored cases. Major postoperative complications were classified as grade 3 (severe adverse events [SAEs] requiring interventional procedures) and grade 4 (life-threatening adverse events requiring a return to the operating theater or intensive care support). Procedure-related mortality was defined as patient death within 30 days after surgery or during the hospital stay (grade 5).

### Statistical Analyses

All statistical analyses were conducted using SPSS Statistics version 24.0 (IBM Corporation, Armonk, NY, USA). All *p* values equal to or lower than 0.05 were considered statistically significant. Quantitative values were reported as mean ± standard deviation (SD) or median (interquartile range [IQR]), and categorical variables as numbers and percentages. Categorical variables were compared between patients with synchronous colorectal PM and those with metachronous colorectal PM using the Chi-square test or Fisher’s exact test. Continuous variables were compared between the two groups using Student’s *t* test or the Mann–Whitney *U* test. Both OS and DFS were compared between the two groups using the log-rank test.

Subsequently, a multivariable Cox regression analysis was performed to determine the impact of metachronous versus synchronous colorectal PM on survival outcomes after adjustment for potential confounders. The potential confounders included were either those with a *p* value lower than 0.20 in the univariate survival analysis or those known from the literature. Results were reported as hazard ratio (HR) with 95% confidence interval (CI).

## Results

### Baseline Characteristics

The study analyzed 433 patients with colorectal PM who underwent CRS with HIPEC. For 231 patients (53%), synchronous colorectal PM was diagnosed, whereas for 202 patients (47%), metachronous colorectal PM after initial curative colorectal resection was diagnosed. Of the patients with synchronous colorectal PM, 202 (87.4%) underwent CRS with HIPEC directly, whereas 29 (12.6%) underwent primary surgery and were referred to one of the tertiary referral hospitals in which CRS with HIPEC was performed in a second stage (Fig. 1).

Table 1 presents the patient characteristics, tumor characteristics, and surgical characteristics of the entire cohort, as well as a comparison of these characteristics between patients with synchronous colorectal PM and those with metachronous colorectal PM. At baseline, the patients with synchronous colorectal PM differed significantly from the patients with metachronous colorectal PM. The patients with metachronous colorectal PM less frequently presented with signet ring cell histology (1.5 vs 11.7%; *p* < 0.001), less frequently had an N2 status (25.2 vs 45.0%; *p* < 0.001), and were less frequently treated with neoadjuvant (14.9% vs 30.3%; *p* < 0.001) or adjuvant (21.8% vs 53.3%; *p* < 0.001) chemotherapy or neoadjuvant biologic therapy (4.5% vs 11.7%; *p* = 0.012). Other baseline characteristics were similar between the two groups.

**Table 1:** Comparison of baseline characteristics between patients with synchronous versus metachronous colorectal peritoneal metastases who underwent CRS with HIPEC

	Total (*n* = 433) *n* (%)	Synchronous colorectal PM (*n* = 231) *n* (%)	Metachronous colorectal PM (*n* = 202) *n* (%)	*p* value
Age (years)	64 ± 10.8	62 ± 11	63 ± 11	0.126
Female sex	224 (51.7)	115 (49.8)	109 (54.0)	0.753
BMI (kg/m^2^)	25.7 ± 4.6	25.8 ± 5.9	25.1 ± 4.7	0.366
ASA				0.688
1	41 (9.5)	23 (10.0)	18 (8.9)	
2	343 (79.2)	181 (78.4)	162 (80.2)	
3	48 (11.1)	27 (11.7)	21 ((10.4)	
4	1 (0.2)	0 (0.0)	1 (0.5)	
Comorbidity				
NIDDM	48 (11.1)	26 (11.3)	22 (10.9)	0.819
IDDM	5 (1.2)	2 (0.9)	3 (1.5)	
Cardiovascular comorbidity	54 (12.5)	28 (12.1)	26 (12.9)	0.338
Hypertension	86 (19.9)	40 (17.3)	46 (22.8)	0.206
Lung comorbidity	13 (3.0)	6 (2.6)	7 (3.5)	0.893
Renal comorbidity	8 (1.8)	3 (1.3)	5 (2.5)	0.611
Primary tumor				0.115
Right colon	149 (34.4)	92 (40.0)	57 (28.2)	
Transverse colon	34 (7.9)	17 (7.4)	17 (8.4)	
Left colon	40 (9.2)	17 (7.4)	23 (11.4)	
Sigmoid	143 (33.0)	66 (28.7)	77 (38.1)	
Rectum	66 (15.2)	38 (16.5)	28 (13.9)	
Signet cell histology	30 (6.9)	27 (11.7)	3 (1.5)	< 0.001
T stage				0.599
≤ 3	184 (42.5)	93 (40.3)	91 (45.0)	
4	216 (49.9)	120 (51.9)	96 (47.5)	
N status				< 0.001
0	119 (27.5)	43 (18.6)	76 (37.6)	
1	126 (29.1)	66 (28.6)	60 (29.7)	
2	155 (35.8)	104 (45.0)	51 (25.2)	
Prior chemotherapy	147 (33.9)	30 (13.0)	117 (57.9)	< 0.001
Prior biological therapy	10 (2.3)	4 (1.7)	6 (3.0)	0.392
Synchronous liver metastases	40 (9.2)	23 (10.0)	17 (8.4)	0.581
Neoadjuvant chemotherapy				< 0.001
Yes	100 (23.1)	70 (30.3)	30 (14.9)	
Neoadjuvant biologic therapy				0.012
Yes	36 (8.3)	27 (11.7)	9 (4.5)	< 0.001
Adjuvant chemotherapy				
Yes	161 (37.2)	120 (53.3)	41 (21.8)	
Adjuvant biologic therapy				0.510
Yes	13 (3.0)	9 (4.0)	4 (2.0)	0.06
PCI at HIPEC (IQR)	8 (4–12)	8.0 (5–12)	7 (3–12)	
HIPEC regimen				0.720
MMC	383 (88.5)	204 (88.3)	179 (88.6)	
Oxaliplatin/5FU/LV	39 (9.0)	22 (9.5)	17 (8.4)	
Cisplantin	1 (0.2)	0 (0.0)	1 (0.5)	
Other regimen	10 (2.3)	5 (2.5)	5 (2.5)	
No. of resections during HIPEC (IQR)	4 (3–6)	4 (3–6)	4 (2–6)	0.139
Operation time (IQR)	383 (312–461)	378 (307–462)	390 (315–460)	0.27
Stoma post-HIPEC	232 (53.6)	125 (54.1)	107 (53.0)	0.812
Resection status				0.590
CC-0 or CC-1	431 (99.5)	230 (99.4)	201 (99.4)	
≥ CC-2	2(0.5)	1 (0.5)	1 (0.5)	
Hospital stay: days (IQR)	13 (8–20)	13 (9–21)	13 (8–20)	0.770
OS: months (95% CI)	34 (30–38)	34 (28–40)	33 (28–38)	0.819
DFS: months (95% Cl)	13 (11–15)	15 (11–19)	11 (10–12)	< 0.001

*CRS* cytoreductive surgery, *HIPEC* hyperthermic intraperitoneal chemotherapy, *PM* peritoneal metastases, *BMI* body mass index (kg/m^2^), *ASA* American Society of Anesthesiologists, *NIDDM* non-insulin-dependent diabetes mellitus, *IDDM* insulin-dependent diabetes mellitus, *PCI* Peritoneal Cancer Index, *IQR* interquartile range, *MMC* Mitomycin-C, *5FU* Fluorouracil, *LV* Leucovorin, *CC score* completeness of cytoreduction score *OS* overall survival *DFS* disease-free survival

### Surgical Morbidity and Mortality

Table 2 presents the mortality and overall postoperative morbidity rates divided by type and severity of the postoperative complication. The number of major postoperative complications was similar between the patients with synchronous colorectal PM and those with metachronous colorectal PM (26.8% vs 29.7%; *p* = 0.693). The perioperative mortality rate for the entire cohort was 1.6% and showed no significant difference between the two groups (*p* = 0.575). The causes of treatment-related death were cardiac events (*n* = 2), major postoperative bleeding (*n* = 2), anastomotic leakage (*n* = 1), and intra-abdominal abscesses (*n* = 2).

**Table 2:** Comparison of major postoperative complications between patients with synchronous versus metachronous peritoneal metastases who underwent CRS with HIPEC

	Synchronous colorectal PM (*n* = 231) *n* (%)	Metachronous colorectal PM (*n* = 202) *n* (%)	*p* value
SAE score			0.693
1–2	70 (30.3)	56 (27.7)	
≥ 3	62 (26.8)	60 (29.7)	
Reoperation	35 (15.2)	30 (14.9)	0.931
Hospital mortality	3 (1.3)	4 (2.0)	0.575
Grade ≥ 3 complications			
Anastomotic leakage	15 (6.5)	16 (7.9)	0.589
Postoperative bleeding	3 (1.3)	2 (1.0)	0.714
Intra-abdominal abscess	28 (12.1)	32 (15.8)	0.379
Wound infection	5 (2.2)	3 (1.5)	0.468
Urinary tract infection	1 (0.4)	2 (1.0)	0.361
Pneumonia	3 (1.3)	4 (2.0)	0.549
Other infection	3 (1.3)	8 (4.0)	0.735
Ileus	6 (2.6)	4 (2.0)	0.630
Gastroparesis	5 (2.2)	6 (3.0)	0.650
Electrolyte disorder	0 (0.0)	1 (0.5)	0.636
Anemia	0 (0.0)	0 (0.0)	1.00
Fistula formation	2 (0.9)	2 (1.0)	0.660
Wound dehiscence	10 (4.3)	7 (3.5)	0.650
Urinoma	4 (1.7)	1 (0.5)	0.286
Pulmonary embolism	1 (0.4)	0 (0.0)	0.338
Cardiac disease	5 (2.1)	3 (1.5)	0.368

*CRS* cytoreductive surgery, *HIPEC* hyperthermic intraperitoneal chemotherapy, *PM* peritoneal metastases, *SAE* serious adverse event

### Survival Outcomes

**Fig. 2:**
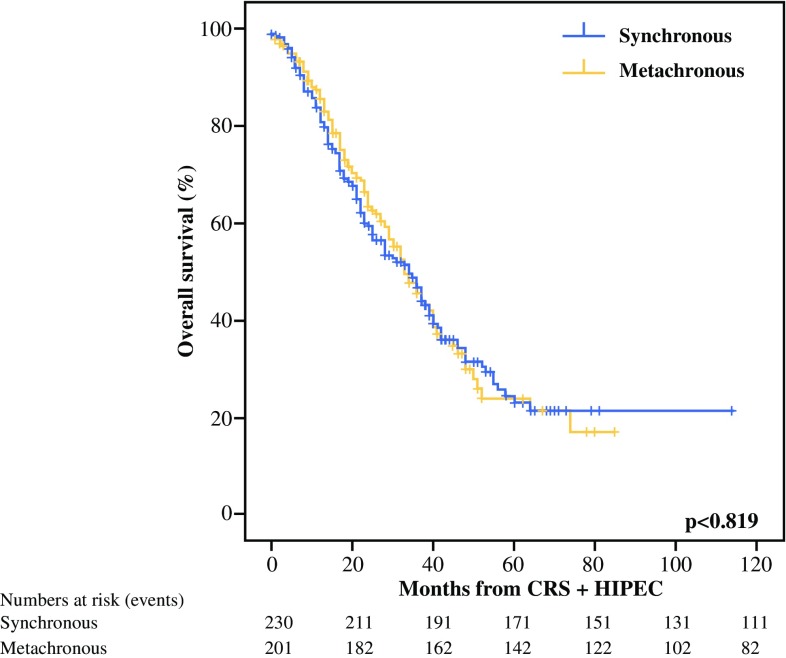
Overall survival of patients with synchronous versus metachronous colorectal peritoneal metastases who underwent CRS with HIPEC

**Fig. 3:**
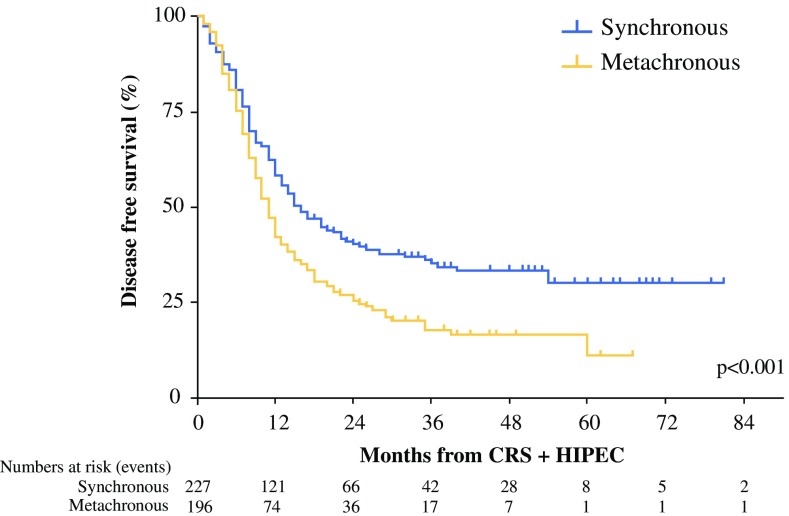
Disease-free survival of patients with synchronous versus metachronous colorectal peritoneal metastases who underwent CRS with HIPEC

In the univariate analysis, the median OS was similar between the patients with synchronous colorectal PM and those with metachronous colorectal PM (34 vs 33 months; *p* = 0.819) (Fig. 2). During the follow-up period, recurrence was diagnosed in 270 patients (62.4%). In the univariate analysis, the median DFS was significantly shorter for the patients with metachronous colorectal PM (11 months; 95% CI 10–12 months) than for the patients with synchronous colorectal PM (15 months; 95% CI 11–19 months) (*p* < 0.001; Fig. 3; Table 3).

**Table 3:** Uni- and multivariable comparison of disease-free survival between patients with synchronous versus metachronous colorectal peritoneal metastases after CRS with HIPEC

Variables	Univariate analysis			Multivariate analysis		
	HR	95% CI	*p* Value	HR	95% CI	*p* Value
Onset of colorectal PM						
Synchronous	1.00	–	–	1.00	–	–
Metachronous	1.51	1.19–1.93	0.001	1.63	1.18–2.26	< 0.01
Age	0.99	0.98–1.00	0.20			
Sex						
Female	1.00	–	–			
Male	1.01	0.79–1.28	0.95			
Primary tumor						
Rectum	1.00	–	–	1.00	–	–
Right colon	0.95	0.65–1.93	0.79	1.00	0.66–1.52	0.99
Transverse colon	0.76	0.44–1.32	0.34	0.75	0.41–1.38	0.35
Left colon	1.05	0.63–1.73	0.86	1.5	0.66–2.00	0.63
Sigmoid	0.91	0.62–1.33	0.62	0.81	0.53–1.23	0.32
Signet cell histology						
No	1.00	–	–	1.00	–	–
Yes	1.23	0.79–1.90	0.36	1.18	0.70–1.99	0.53
PCI score during CRS with HIPEC						
0–5	1.00	–	–	1.00	–	–
6–10	1.47	1.07–2.04	0.02	1.33	0.96–1.88	0.09
11–15	2.06	1.42–2.99	< 0.001	2.05	1.38–3.07	< 0.001
16–20	1.99	1.27–3.11	< 0.01	1.94	1.22–3.09	< 0.01
> 20	2.00	0.99–4.02	0.05	2.28	1.10–4.71	0.03
CC score						
CC-0 or CC-1	1.00	–	–			
CC ≥ 2	3.84	0.54–27.58	0.18			
Prior chemotherapy						
No	1.00	–	–	1.00	–	–
Yes	1.41	1.10–1.81	< 0.01	1.07	0.78–1.47	0.67
Neoadjuvant chemotherapy (CRS with HIPEC)						
No	1.00	–	–			
Yes	0.99	0.74–1.32	0.93			
Adjuvant chemotherapy (CRS with HIPEC)						
No	1.00	–	–	1.00	–	–
Yes	0.63	0.54–0.81	< 0.001	0.72	0.54–0.97	0.03
Neoadjuvant biologic therapy (CRS with HIPEC)						
No	1.00	–	–			
Yes	1.20	0.76–1.89	0.44			

*HR* hazard ratio, *CI* confidence interval, *PM* peritoneal metastases, *PCI* Peritoneal Cancer Index, *CRS* cytoreductive surgery, *HIPEC* hyperthermic intraperitoneal chemotherapy, *CC score* completeness of cytoreduction score

In the multivariate analysis, adjusted for tumor location, signet cell histology, PCI score, resection status, prior chemotherapy, and adjuvant chemotherapy after CRS with HIPEC, metachronous colorectal PM was associated with a worse DFS than synchronous colorectal PM (adjusted HR 1.63; 95% CI 1.18–2.26; *p* < 0.01) (Table 3). The location of recurrent disease was available for 242 patients and included colorectal PM only (*n* = 113, 46.7%), colorectal PM and distant metastases (*n* = 70, 28.9%), and distant metastases only (*n* = 59, 24.4%). Organ-specific locations of the distant metastases were most likely the liver (*n* = 62, 48.0%), the lung (*n* = 43, 33.3%), or both organs simultaneously (*n* = 20, 15.5%). The location of recurrent disease did not differ significantly between the two groups (*p* = 0.482).

**Fig. 4:**
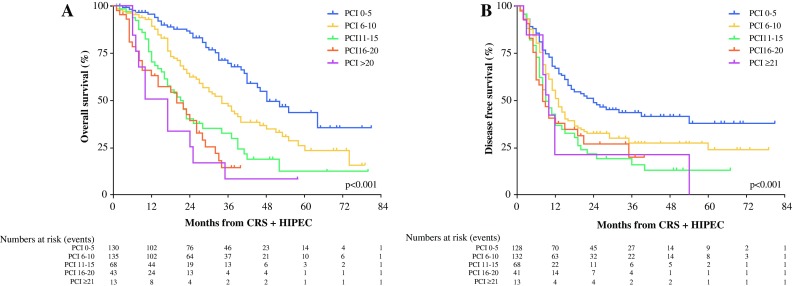
Kaplan–Meier survival curves for all 433 patients according to Peritoneal Cancer Index (PCI) score. **a** Overall survival (OS). **b** Disease-free survival (DFS)

The OS and DFS for all 433 patients according to the PCI score are shown in Fig. 4a, b. The PCI scores were categorized into five different subgroups. A lower PCI score at the time of exploratory laparotomy was associated with a better OS and DFS (*p* < 0.001).

### Additional Analyses of Patients with Metachronous Colorectal PM

The patients with metachronous colorectal PM had a significantly shorter DFS than the patients with synchronous colorectal PM after CRS with HIPEC, without a difference in OS. Further analyses were deemed necessary to find an explanation for this difference, and to identify which specific metachronous colorectal PM patient is at risk for a decreased DFS after CRS with HIPEC.

The group of patients with metachronous colorectal PM in our cohort appeared to be very heterogeneous. We performed a subanalysis, comparing metachronous cancer patients with early (< 1 year) and late (≥ 1 year) recurrences after CRS with HIPEC (Table S1).

The mean OS was significantly shorter for the early recurrence group (19 months; 95% CI 16–21 months) than for the patients who had a late recurrence (30 months; 95% CI 26–35 months; *p* < 0.001). At baseline, the patients who had metachronous colorectal PM with early recurrence differed significantly from the patients with late recurrence. The patients with an early recurrence had a shorter period between primary surgery and onset of metachronous colorectal PM (*p* = 0.017*)*, a higher PCI score (*p* < 0.001), a longer surgery (422 vs 352 min; *p* < 0.001), and more blood loss (800 vs 600 ml; *p* = 0.008) during CRS with HIPEC, which was accompanied by more major postoperative complications (31.2% vs 24.4%; *p* = 0.005) and a longer hospital stay (14 vs 11 days; *p* = 0.002) (Table S1). We adjusted for these potential cofounders in the multivariate regression analyses.

The PCI score had a significant impact on OS and DFS for all 433 patients. We performed additional analyses to identify a possible cutoff point for the PCI score of the patients with metachronous colorectal PM for performing CRS with HIPEC regarding OS and DFS. The PCI scores of the 202 patients with metachronous colorectal PM were divided into the following five different subgroups: PCI of 0–5, PCI of 6–10, PCI of 11–15, PCI of 16–20, and PCI higher than 20. The median OS in the different subgroups was respectively 46 months (95% CI 39–53 months), 34 months (95% CI 22–46 months), 20 months (95% CI 15–25 months), 22 months (95% CI 9–35 months), and 10 months (95% CI 6–14 months). The DFS in the different subgroups was respectively 17 months (95% CI 10–24 months), 11 months (95% CI 9–14 months), 9 months (95% CI 7–12 months), 8 months (95% CI 4–12 months), and 9 months (95% CI 7–11 months).

## Discussion

This prospective observational study that included 433 patients with colorectal PM showed that the patients with metachronous PM had a worse median DFS than the patients with synchronous PM after CRS with HIPEC, whereas OS and surgical morbidity were similar between the two groups.

Currently, most available prognostic factors for survival after CRS with HIPEC are determined in the operating theater. However, these factors cannot be used preoperatively during multidisciplinary HIPEC meetings when clinicians are assessing which patient will benefit from this major procedure. The impact of the time when the colorectal PM developed might be of relevance in predicting outcomes. Synchronous onset of PM might be considered as a proof of aggressive presentation. However, our finding that patients with synchronous PM have an increased DFS contradicts this theory.

On the other hand, metachronous PM could be seen as a proof of the recurrent character of the disease, especially when there is little time between the first tumor and the finding of colorectal PM. However, substantial knowledge and scientific evidence of the impact on survival is lacking.

Currently only three studies have reported the impact that the onset of colorectal PM has on OS.^19^^,^^31^^,^^32^ The data of these three studies (319 patients) were combined in a meta-analysis, in which the pooled HR demonstrated that onset of PM has no effect on OS (HR 1.21; 95% CI 0.87–1.68; *p* = 0.25), comparable with our results.^22^ None of these studies reported on DFS.

In our cohort, the patients with synchronous colorectal PM more frequently received neoadjuvant and adjuvant chemotherapy around the CRS with HIPEC procedure than the patients with metachronous colorectal PM. It could be argued that this led to the difference in DFS after CRS with HIPEC between the two groups.

First, an explanation for the difference in frequencies could be that most metachronous patients experience PM shortly after primary resection and adjuvant chemotherapy (data not shown). Development of PM shortly after the use of chemotherapy can cause the HIPEC surgeon to decide to perform CRS with HIPEC without using neoadjuvant chemotherapy because the patient already is experiencing progression of peritoneal disease shortly after the use of chemotherapy.

We looked at the impact of perioperative chemotherapy on DFS in our multivariate analyses. Only the use of adjuvant chemotherapy was associated with an increase in DFS, but the onset of colorectal PM (synchronous or metachronous) remained an independent risk factor for a decreased DFS. Despite the widespread use of perioperative systemic chemotherapy, no randomized studies have investigated its impact on survival outcomes after CRS with HIPEC, leading to controversy regarding its efficacy, timing, and risks. Consequently, no worldwide consensus exists on the use and timing of perioperative chemotherapy, which varies considerably between HIPEC centers.^33^ We hope that the CAIRO 6 trial, a multicenter, open-label, phases 2 and 3 randomized controlled trial (RCT), will provide some answers about the oncologic efficacy of perioperative systemic therapy and CRS with HIPEC versus upfront CRS with HIPEC (control arm) for isolated resectable colorectal PM (NCT02758951).

The clinical relevance of the finding that the metachronous colorectal PM patients had earlier recurrences than the synchronous colorectal PM patients, without a difference in OS, raises many questions. Most metachronous colorectal PM patients undergo their primary colorectal tumor resection and experience their first recurrence several months later (e.g. colorectal PM > 6 months later). Subsequently, after undergoing CRS with HIPEC, the patients in this cohort had their second recurrence after a median of 11 months while most were still recovering from this major surgical procedure.^34^–^40^ Although OS between synchronous versus metachronous colorectal PM was still comparable, we suspect that the quality of life (QoL) in the months after the second recurrence for the patients with metachronous colorectal PM after CRS with HIPEC might be poor and can therefore not be compared with their synchronous counterparts who are still without a recurrence at this stage.^34^^,^^41^

Qualitative data about the true impact of CRS with HIPEC on different life domains of QoL are still lacking. At this writing, we are performing semi-structured interviews with patients before and 3 months after CRS with HIPEC to identify its true impact on different life domains because we suspect it will contribute to the discussion about QoL of life after CRS with HIPEC.

The group of patients with metachronous colorectal PM in our cohort appeared to be very heterogeneous. Evaluating the data of our multivariate regression analysis, it seems that these patients had a tumor with variable pathogenesis (Table S1). The mean OS was significantly shorter for the early recurrence group (19 months; 95% CI 16–21 months) than for the patients who had a late recurrence (30 months; 95% CI 26–35 months; *p* < 0.001). This result is comparable with that of previous studies, which showed early recurrence after CRS with HIPEC to be associated with a decrease in OS.^1^^,^^19^^,^^21^^,^^26^^,^^42^–^45^ These findings illustrate the difficulty of predicting early recurrence after CRS with HIPEC.

### New Avenues for Research

In our total cohort, the average DFS was only 13 months after CRS with HIPEC despite achievement of complete macroscopic CRS in 431 patients (99.5%). This indicates that the outcomes of CRS with HIPEC might be further improved only if we focus on microscopic (invisible) disease. Local recurrence or colorectal PM will be caused in particular by insufficient treatment of microscopic disease and aggressive tumor biology rather than by macroscopic visible peritoneal disease. For example, several studies have identified four molecular subtypes among patients with colorectal tumors, called the Consensus Molecular Subtypes (CMS1 to CMS4).^46^–^49^ In particular, CMS4 represents highly aggressive tumors, which have been associated with worse DFS and OS. Tumor biology could be an additional selection criterion for CRS with HIPEC in the future.

High recurrence rates after CRS with HIPEC also could be caused by misinterpretation of the completeness of cytoreduction by the HIPEC surgeons. Surgeons still rely on visual and tactile inspection for intraoperative differentiation between tumor and benign tissue to reach a complete cytoreduction. A clear need exists for an intraoperative imaging technique to improve tumor detection.

In recent years, optical molecular imaging using tumor-targeted fluorescence tracers has emerged as a promising real-time imaging technique to improve tumor detection.^50^–^52^ The first phase 1 clinical trials have been performed.^53^^,^^54^ Although no conclusions can be drawn to date with regard to the impact on clinical decision-making, it appears that molecular fluorescence-guided surgery has the potential to help identify tumor tissue during DLS and to attain a more complete cytoreduction during CRS with HIPEC.

### Study Strengths and Limitations

The current study included a relatively large sample. Follow-up evaluation between the patients with synchronous colorectal PM and those with metachronous colorectal PM did not differ and could therefore not explain the difference in DFS. Although data were prospectively maintained, some were missing, which may have had an impact on the results of this study. The patients included in this study underwent surgery in two highly experienced and high-volume HIPEC centers. Thus, our results might not be generalisable to other medical centers.

We should take into account that the patients with synchronous colorectal PM more frequently had signet cell histology than those with metachronous colorectal PM. Moreover, they more frequently had an N2 status and were more frequently treated with neoadjuvant and adjuvant chemotherapy. However, we adjusted for these potential cofounders in the multivariate regression analysis, and the development of metachronous colorectal PM remained a significant independent risk factor for reduced DFS.

## Conclusions

Patients with metachronous colorectal PM have a worse DFS after CRS with HIPEC than patients with synchronous colorectal PM, whereas OS and surgical morbidity are similar between the two groups. Therefore, we recommend extra carefulness in the selection of patients with metachronous colorectal PM who have a PCI above 10 for CRS with HIPEC because of the markedly worse OS and DFS in this specific group of patients. Therefore, next to other risk factors for a worse outcome, time to onset of colorectal PM development should be taken into consideration to optimize patient selection for this major procedure.

## Electronic supplementary material

Below is the link to the electronic supplementary material.

Supplementary material 1 (DOCX 21 kb)
