# Supplementary material for: Impact of Synchronous Versus Metachronous Onset of Colorectal Peritoneal Metastases on Survival Outcomes After Cytoreductive Surgery (CRS) with Hyperthermic Intraperitoneal Chemotherapy (HIPEC): A Multicenter, Retrospective, Observational Study
Source: Ann Surg Oncol. 2019 Mar 15;26(7):2210–21. doi: 10.1245/s10434-019-07294-y (PMC6545176; doi:10.1245/s10434-019-07294-y)
Supplement: Supplementary file 1 — Supplementary material 1 (DOCX 21 kb) [file 10434_2019_7294_MOESM1_ESM.docx]

**Table S1** – Baseline characteristics according to early and late recurrence after CRS with HIPEC in patients with metachronous peritoneal metastases

|  | | | **Early recurrence**  **n = 112** | **Late recurrence**  **n = 86** | *P-value* |
| --- | --- | --- | --- | --- | --- |
| Age, y ± SD | | | 65 ± 10 | 65 ± 11 | 0.271 |
| Female sex, n (%) |  | | 62 *(55.4)* | 46 *(53.5)* | 0.826 |
| ASA, n (%)  1  2  3  4 |  | | 10 *(8.9)*  92 *(82.1)*  9 *(8.0)*  1 *(0.9)* | 8 *(9.3)*  67 *(77.9)*  11 *(12.8)*  0 *(0.0)* | 0.712 |
| Smoking, n (%) |  | | 21 *(18.8)* | 23 *(26.7)* | 0.704 |
| Synchronous liver metastases, n (%) |  | | 11 *(9.8)* | 6 *(7.0)* | 0.139 |
| Interval primary surgery to mPM, months ±SD | |  | 17 ± 15 | 18 ± 29 | **0.017** |
| Neoadjuvant chemotherapy | | | 14 *(12.5)* | 14 *(16.3)* | 0.104 |
| Adjuvant chemotherapy | | | 19 *(17.0)* | 22 *(25.6)* | 0.129 |
| T4 primary tumor | | | 47 *(42.0)* | 48 *(55.8)* | 0.059 |
| Perforated primary tumor | | | 3 *(2.7)* | 6 *(7.0)* | 0.156 |
| PCI at HIPEC, n (%)  0-5  6-10  11-15  16-20  >21  Unknown  Operation time, min ± SD | | | 28 *(25.0)*  36 *(32.1)*  22 *(19.6)*  11 *(11.6)*  4 *(3.6)*  9 (8.0)  422 ± 106 | 40 *(46.1)*  28 *(32.6)*  7 *(8.1)*  4 *(4.7)*  2 *(2.3)*  5 *(5.8)*  352 ± 125 | **<0.001**  **<0.001** |
| Blood loss, ml ± SD | | | 800 ± 1038 | 600 ± 1139 | **0.008** |
| Stoma post HIPEC | | | 62 *(55.4)* | 43 *(50.0)* | **0.**741 |
| Resection status  CC-0 or CC-1  ≥CC-2 | | | 112 *(100)*  0 *(0.0)* | 86 *(100)*  0 *(0.0)* | 0.950 |
| Length of hospital stay, days ± SD | | | 14 ± 15 | 11 ± 16 | **0.002** |
| SAE score  1-2  ≥3 | | | 37 *(33.1)*  35 *(31.2)* | 19 *(22.1)*  21 *(24.4)* | **0.005** |
| Re-operation, n (%) | | | 18 *(16.1)* | 11 *(12.8)* | 0.092 |
| OS, months (95% Cl) | | | 19 (16-21) | 30 (26-35) | **<0.001** |
|  | | |  |  |  |

*SD* standard deviation, *ASA* american society of anesthesiologists, *mPM* metachronous peritoneal metastases, *PCI* peritoneal cancer index, *HIPEC* hyperthermic intraperitoneal chemotherapy, *R-score* completeness of cytoreductive score, *SAE* serious adverse event, OS overall survival. Values in parentheses are percentages.
